# Prognosis of Cervical Cancer in the Era of Concurrent Chemoradiation from National Database in Korea: A Comparison between Squamous Cell Carcinoma and Adenocarcinoma

**DOI:** 10.1371/journal.pone.0144887

**Published:** 2015-12-14

**Authors:** Jung-Yun Lee, Young Tae Kim, Sunghoon Kim, Boram Lee, Myong Cheol Lim, Jae-Weon Kim, Young-Joo Won

**Affiliations:** 1 Department of Obstetrics and Gynecology, Institute of Women’s Life Medical Science, Yonsei University College of Medicine, Seoul, Korea; 2 Cancer Registration and Statistics Branch, National Cancer Center, Goyang, Korea; 3 Gynecologic Cancer Branch & Center for Uterine Cancer, National Cancer Center, Goyang, Korea; 4 Department of Obstetrics and Gynecology, Seoul National University College of Medicine, Seoul, Korea; University of Florida, UNITED STATES

## Abstract

In 1999, the National Cancer Institute issued a clinical advisory strongly touting the advantage of cisplatin-based chemoradiation (CCRT) for cervical cancer patients requiring radiation for their treatment. This study aimed to compare survival outcomes of cervical squamous cell carcinoma and adenocarcinoma before and after the advent of CCRT. Data were obtained from the Korea National Cancer Incidence Database for patients who were diagnosed with cervical cancers between 1993 and 2012. We compared survival according to histologic subtypes in cervical cancer patients diagnosed before (1993–1997), during (1998–2002), and after (2003–2012) the introduction of CCRT. A total of 80,766 patients were identified, including 64,531 (79.9%) women with squamous cell carcinomas and 7,265 (9.0%) with adenocarcinoma. With the introduction of CCRT, survival trends gradually increased in patients of both histologic subtypes with regional tumors. However, survival was significantly higher in squamous cell carcinoma than in adenocarcinoma patients regardless of treatment modalities (surgery alone, P < 0.001; surgery followed by CCRT, P < 0.001; or primary CCRT, P = 0.003). Multivariate analysis showed that adenocarcinoma was an independent negative prognostic factor for survival regardless of the time period (before CCRT, hazard ratio (HR) = 1.49; 95% confidence interval (CI), 1.37–1.62; after introduction of CCRT, HR = 1.40; 95% CI, 1.30–1.50). Although the survival of adenocarcinoma has improved after the introduction of CCRT, adenocarcinoma is still associated with worse overall survival compared to squamous cell carcinoma in the era of CCRT.

## Introduction

Although the incidence and mortality rate for cervical cancer has been decreasing in recent years, it continues to be a major public health problem worldwide including in East Asia [1–3]. In contrast to a marked decrease in the incidence of squamous cell carcinoma of the cervix, that of adenocarcinoma has been stable or even increasing [4, 5]. As the current guidelines for cervical cancer recommend the same treatment regardless of histologic subtypes, increasing efforts have focused on comparing the prognoses of adenocarcinoma to squamous cell carcinoma.

Previous studies have evaluated the prognostic role of tumor histology on cervical cancer outcomes with conflicting results [6–9]. Many of these studies included cohorts that were small and from single institutions, or included patients treated over long periods of time. Using a large database, Galic et al. concluded that adenocarcinoma negatively impacts survival outcome regardless of whether histology shows early or advanced stage disease [6].

In 1999, the National Cancer Institute issued an advisory urging clinicians to strongly consider the use of cisplatin-based concurrent chemoradiation (CCRT) to treat cervical cancer patients for whom radiation treatment was indicated [10]. Since then, CCRT has been widely used as a primary or adjuvant treatment option instead of radiation therapy alone in developed countries [11]. Because of the survival benefits of CCRT, it is prudent to compare its efficacy in individual histologic subtypes of cervical cancer. Therefore, the aim of this study was to compare the historical changes in survival trends of squamous cell carcinoma versus adenocarcinoma patients before, during, and after the introduction of CCRT using data from the Nationwide Cancer Registry. Additionally, we compared overall survival outcomes between the two histologic subtypes in recent years since the dissemination of CCRT.

## Patients and Methods

We analyzed the cervical cancer data from the Korea Central Cancer Registry (KCCR). The ministry of Health and Welfare initially launched the KCCR as a nationwide, hospital-based cancer registry in 1980. The KCCR covers the entire population under the Population-Based Regional Cancer Registry program since 1999 [12]. Furthermore, the Gynecologic Oncology Committee of the Korean Society of Obstetrics and Gynecology has operated a gynecologic cancer registry since 1991 [4]. Using these two databases, we could estimate the national cervical cancer incidences since 1993. We included 72,240 cases from our previous study published in 2013 [13].

Demographic data collected included age at diagnosis (<40, 40–49, 50–59, and ≥60 years). Patients were classified based on their tumor histology into the following groups: squamous, adenocarcinoma, and adenosquamous carcinoma. Staging information was based on the Surveillance, Epidemiology, and End Results (SEER), summary staging (localized, regional, distant), and Féderation Internationale de Gynécologie et d’Obstétrique (FIGO). Summary staging is a basic method of categorizing how far a cancer has spread from its origin [14]. Several cancer registries in North America report their data by the summary stage, as the staging categories are broad enough to measure the success of cancer control and other epidemiologic efforts [15]. For consistency, the FIGO stage was converted to the SEER stage. The year of diagnosis was categorized into one of the following ranges: 1993–1997 (before CCRT), 1998–2002 (during CCRT rollout), 2003–2007, and 2008–2012 (both after CCRT).

The National Cancer Center Institutional Review Board approved this study (NCC2015-0135). The data were obtained and analyzed after anonymized and de-identified.

Age-standardized rates (ASRs) were calculated using the world standard population [16]. Survival analysis was according to 5-year relative survival rates. The relative survival is an estimate based on the ratio between the overall survival in a group of patients and overall survival in a similarly sized group from the general population with same age distribution and without disease [17]; the details of this method were described previously [18]. We compared the survival according to histologic subtypes in cervical cancer patients diagnosed before, during, and after introduction of CCRT. In Korea, CCRT was adopted as routine practice between 1998 and 2002, and has been commonly performed since 2003 (S1 Table). Categorical variables were compared with the Pearson chi-square test. The Cox proportional hazards model was used to evaluate independent prognostic factors and to estimate their covariate-adjusted effects on relative survival. This model assumes that additive changes in the value of a survival variable cause corresponding multiplicative changes in the hazard function. All analyses were performed with SAS version 9.3 (SAS Institute Inc., Cary, NC, USA).

## Results

Table 1 shows the demographic characteristics of the study population. A total of 80,766 patients were identified, including 64,531 (79.9%) women with squamous cell carcinomas, 7,265 (9.0%) with adenocarcinoma, and 1,853 (2.3%) with adenosquamous carcinomas. Primary treatment consisted of surgery in 33,012 (40.9%) patients, followed by primary concurrent chemoradiation in 7,216 (8.9%) of the patients encompassing all histologic subtypes.

**Table 1:** Patient characteristics.

	No. of cases	%
Age (years)		
<40	16,913	20.9
40–49	22,207	27.5
50–59	17,803	22.0
≥60	23,843	29.5
Stage		
Localized	33,609	41.6
Regional	17,757	22.0
Distant	2,786	3.5
Unspecified	26,614	33.0
Year of Diagnosis		
1993–1997	21,688	26.9
1998–2002	21,759	26.9
2003–2007	19,222	23.8
2008–2012	18,097	22.4
Primary treatment		
Surgery	33,012	40.9
Chemotherapy	6,933	8.6
Radiation	6,395	7.9
Chemoradiation	7,216	8.9
Surgery + adjuvant chemoradiation	4,647	5.8
Others	22,563	27.9
Histology		
Squamous cell carcinoma	64,531	79.9
Adenocarcinoma	7,265	9.0
Adenosquamous carcinoma	1,853	2.3
Unspecified	7,117	8.8

**Fig 1:**
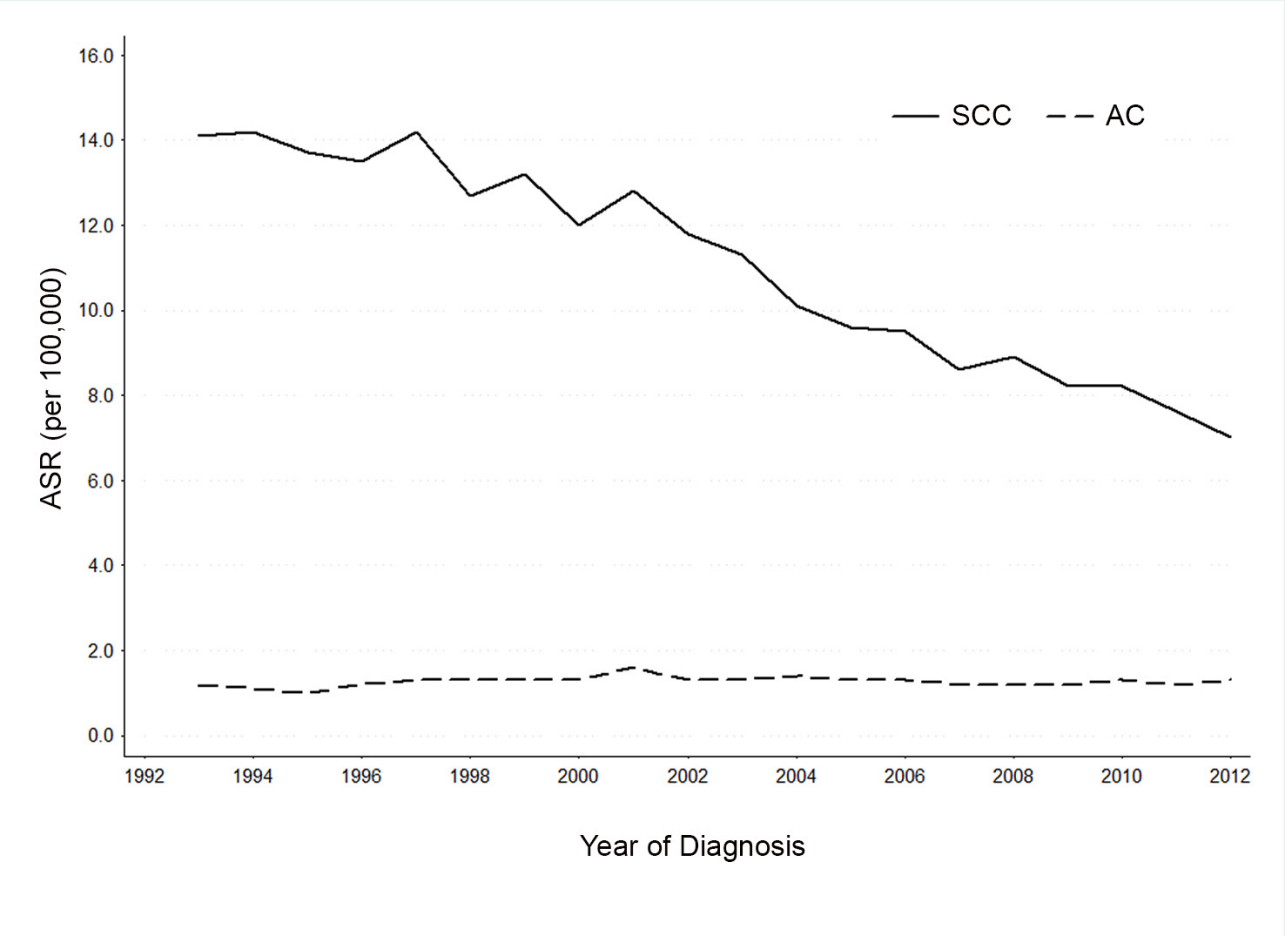
Cervical cancer incidence trends by tumor histology.

While the overall incidence rate of adenocarcinoma remained relatively constant from 1993 to 2012, that of squamous cell carcinoma declined during the same period of time (Fig 1). The age-standardized incidence rates of adenocarcinoma were in the range of 1.2–1.3 per 100,000, while those of squamous cell carcinoma declined from 14.1 to 7.0 per 100,000 during the study period.

Table 2 shows the 5-year relative survival rate (RSR) of patients with cervical cancer according to histologic subtypes and SEER stage. During this period, the overall 5-year RSR was not significantly changed for either squamous cell carcinoma or adenocarcinoma. However, differences in survival were observed when comparing specific time periods. There was a decrease (3%) in the 5-year RSR of localized squamous cell carcinoma patients diagnosed in 2008–2012 compared to that of those diagnosed in 1993–1997. From 1993 to 2012, survival gradually increased for patients with regional tumors; the 5-year RSR improved from 64.5% (1993–1997) to 75.1% (2008–2012) for regional squamous cell carcinoma, corresponding to an increase of 10.6% from 1993 to 2012 (P < 0.001). Furthermore, there was a significant increase in the 5-year RSR for regional adenocarcinoma, corresponding to 9.1% between 1993 and 2012 (P = 0.027).

**Table 2:** Five-year relative survival rate classified by tumor histology.

	Year of Diagnosis				Difference*	P value
	1993–1997	1998–2002	2003–2007	2008–2012		
Squamous cell carcinoma	81	82.7	83	82	1	0.754
Localized	95.2	95.7	93.4	92.2	-3	**< .001**
Regional	64.5	69.9	72.4	75.1	10.6	**< .001**
Distant	21.8	25	30	28.8	7	0.055
Adenocarcinoma	73	79	79.1	78.2	5.2	0.073
localized	89.8	92	90.9	91.3	1.5	0.911
regional	53.7	56.6	65.5	62.8	9.1	**0.027**
distant	0	24.1	27.3	24.2	24.2	0.863

*percent change in the 5-year relative survival rate of patients diagnosed in 2008–2012 compared to those diagnosed in 1993–1997

**Fig 2:**
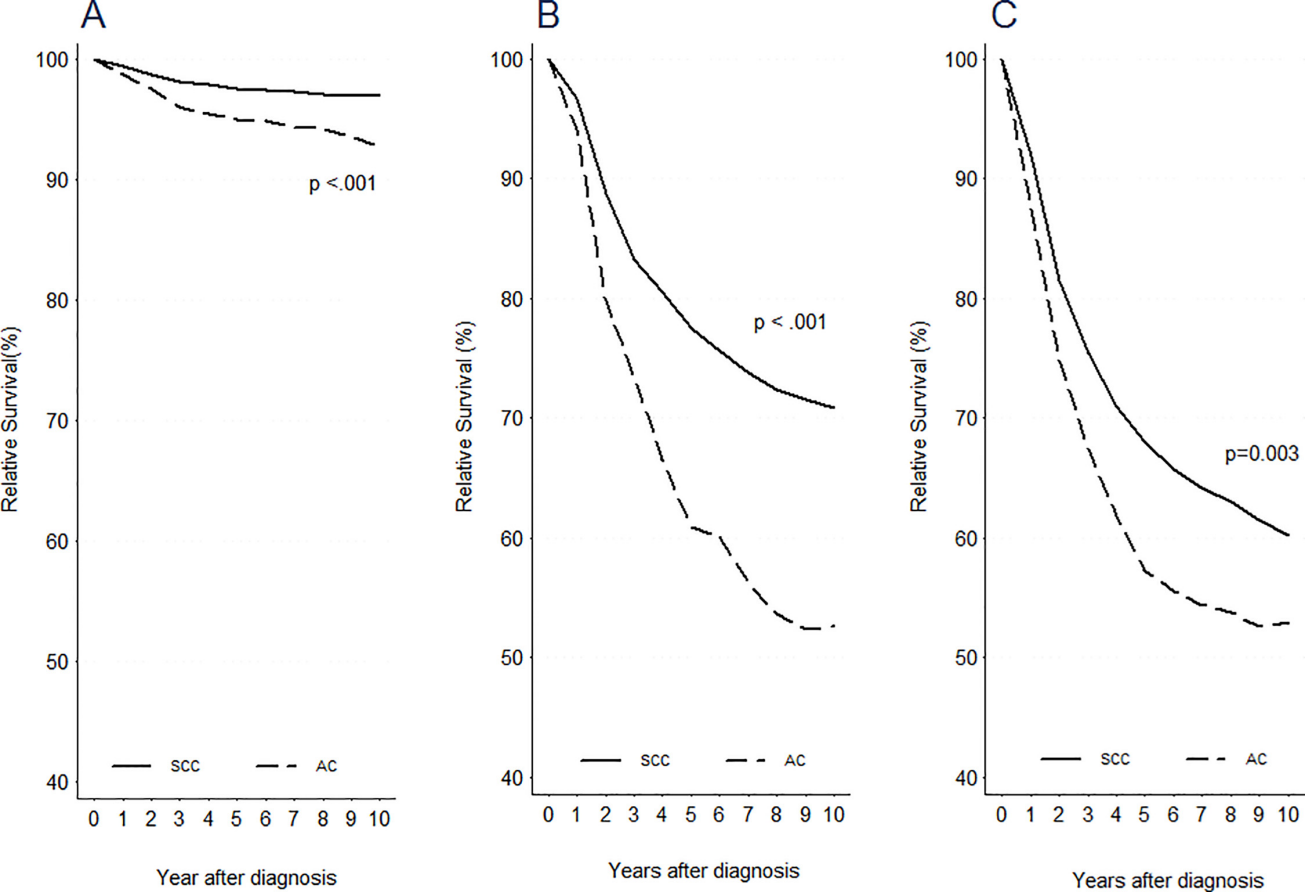
Survival curves for patients according to primary treatment classified by tumor histology after (2003–2012) the introduction of chemoradiation. (A) Surgery alone, (B) Surgery followed by adjuvant chemoradiation, (C) Primary chemoradiation.

Relative survival was significantly higher in squamous cell carcinoma than in adenocarcinoma regardless of treatment modalities (Fig 2). When treated with surgery alone, patients with adenocarcinoma (n = 1,905) showed a poorer survival outcome compared to those with squamous cell carcinoma (n = 12,965) (P < 0.001). When treated with surgery followed by adjuvant CCRT, patients with adenocarcinoma (n = 474) showed a significantly poorer survival compared to those with squamous cell carcinoma (n = 2,682) (P < 0.001). Moreover, CCRT-treated patients with adenocarcinoma (n = 302) had poorer survival compared to those with squamous cell carcinoma (n = 4,245) (P = 0.003).

Cox multivariate modeling was used to analyze prognostic factors for overall-survival in all patients, controlling for other variables (Table 3). Adenocarcinoma was an independent negative prognostic factor for survival regardless of the time period. Before the CCRT era (1993–1997), patients with adenocarcinoma were 49% more likely to die than those with squamous cell carcinoma (hazard ratio [HR] = 1.49; 95% confidence interval [CI], 1.37–1.62). After the introduction of CCRT, histology continued to be a factor determining survival. Patients with adenocarcinoma were 40% more likely to die than those with squamous cell carcinoma (HR = 1.40; 95% CI, 1.30–1.50).

**Table 3:** Estimated hazard ratio of cervical cancer before, during, and after the introduction of CCRT.

	Total		1993–1997		1998–2002		2003–2012	
	N	Adj HR	N	Adj HR	N	Adj HR	N	Adj HR
Age (years)								
<40	16,913	0.84 (0.80–0.88)	5,122	0.76 (0.70–0.83)	4,665	0.85 (0.77–0.93)	7,126	0.94 (0.86–1.03)
40–49	22,207	ref	5,652	ref	6,205	ref	10,350	ref
50–59	17,803	1.35 (1.29–1.40)	5,468	1.48 (1.38–1.59)	4,572	1.42 (1.31–1.53)	7,763	1.14 (1.06–1.23)
≥60	23,843	2.74 (2.64–2.84)	5,446	2.78 (2.61–2.97)	6,317	2.99 (2.79–3.21)	2,080	2.48 (2.33–2.64)
Stage								
Localized	33,609	ref	6,322	ref	7,797	ref	19,490	ref
Regional	17,757	2.13 (2.04–2.21)	4,105	2.29 (2.13–2.47)	4,500	2.18 (2.01–2.36)	9,152	1.96 (1.85–2.09)
Distant	2,786	6.73 (6.36–7.12)	268	6.51 (5.65–7.51)	417	6.98 (6.15–7.92)	2,101	6.59 (6.13–7.09)
Year of Diagnosis								
1993–1997	21,688	1.05 (1.02–1.09)	21,688	-	-	-	-	-
1998–2002	21,759	ref	-	-	21,759	-	-	-
2003–2007	19,222	1.03 (0.99–1.07)	-	-	-	-	19,222	ref
2008–2012	18,097	0.99 (0.95–1.04)	-	-	-	-	18,097	1.00 (0.96–1.05)
Primary treatment								
Surgery	33,012	ref	8,032	ref	8,600	ref	16,380	ref
Chemotherapy	6,933	3.74 (3.55–3.94)	3,044	3.27 (3.02–3.54)	2,110	3.41 (3.10–3.76)	1,779	4.77 (4.31–5.29)
Radiation	6,395	4.07 (3.87–4.28)	2,584	3.63 (3.36–3.93)	1,975	3.60 (3.28–3.95)	1,836	5.15 (4.69–5.68)
Chemoradiation	7,216	3.58 (3.39–3.78)	779	4.27 (3.83–4.75)	1,490	3.55 (3.20–3.93)	4,947	4.07 (3.73–4.43)
Surgery + adjuvant chemoradiation	4,647	3.44 (3.22–3.68)	309	4.33 (3.69–5.08)	748	4.05 (3.56–4.60)	3,590	3.73 (3.39–4.11)
Others	22,563	3.67 (3.51–3.83)	6,940	2.96 (2.76–3.18)	6,836	3.29 (3.05–3.56)	8,787	5.02 (4.65–5.42)
Histology								
Squamous cell carcinoma	64,531	ref	17,623	ref	17,663	ref	29,245	ref
Adenocarcinoma	7,265	1.44 (1.37–1.51)	1,454	1.49 (1.37–1.62)	1,866	1.45 (1.33–1.58)	3,945	1.40 (1.30–1.50)
Adenosquamous carcinoma	1,853	1.26 (1.15–1.37)	438	1.20 (1.03–1.40)	488	1.39 (1.18–1.63)	927	1.23 (1.01–1.41)

HR, hazard ratio; ref, reference

## Discussion

This large population-based epidemiologic study showed that survival improved in regional tumors after the introduction of CCRT. However, adenocarcinoma was still an independent negative prognostic factor despite the availability of CCRT. Adenocarcinoma had poorer survival than squamous cell carcinoma both before and after the introduction of CCRT, suggesting that histologic subtypes have an important impact on survival for women with cervical cancer.

In this study of 80,766 cases of cervical cancer in Korea, there was an increase in adenocarcinoma incidence cases from 6.7% in 1993–1997 to 11.2% in 2008–2012. Recent reports show that the relative and absolute incidences of adenocarcinoma have risen, and now account for approximately 20% of invasive cervical cancer [5, 19]. This may be due to less success in the diagnosis and treatment of pre-invasive adenocarcinoma compared to squamous cell carcinoma [20–23]. Given the relative rise in the incidence of adenocarcinoma, its prognostic significance is of particular importance.

It remains controversial in the field whether the histologic subtype is an independent prognostic factor for cervical cancer. For conclusive study, an appropriate number of adenocarcinoma cases should be analyzed while controlling for stage and primary treatment. As sensitivity to radiotherapy has been proposed to vary according to histology [24], our analysis specifically explored the influence of histologic subtypes, stratified by primary treatment and time period, on outcome.

Rose et al. compared squamous cell carcinoma, adenocarcinoma, and adenosquamous cell carcinoma outcomes according to treatment modality [8]. They showed that adenocarcinoma/adenosquamous carcinoma (n = 182) are associated with worse outcome when treated with radiation alone, but outcome was similar to squamous cell carcinoma when treated with CCRT. In contrast, we separated adenocarcinoma and adenosquamous carcinoma and showed that adenocarcinoma persists as a negative independent prognostic factor even after introduction of CCRT. Galic et al. performed a sensitivity analysis before (1988–1999) and after (2000–2005) the introduction of chemoradiation [6]. They showed that adenocarcinoma patients had significantly lower survival even after adjustment for other demographic and treatment characteristics. We confirmed that adenocarcinoma had worse overall survival than squamous cell carcinoma regardless of the treatment modality and time period.

This large population-based analysis may reflect the clinical scenarios in other developed countries where CCRT has become widely available. For regional tumors, primary or adjuvant CCRT are commonly indicated, and survival benefits have been shown in both histologic subtypes. However, patients with adenocarcinoma are still 40% more likely to die than those with squamous cell carcinoma. Hence, adenocarcinoma is probably a distinct clinical entity from squamous cell carcinoma and is fundamentally different at the molecular level.

Some investigators have suggested that the molecular mechanisms for the pathogenesis of adenocarcinoma and squamous cell carcinoma are distinct. Significant differences in gene expression were observed between the two histologic subtypes [25]. Different patterns of *P53* mutations were found in squamous cell carcinoma and adenocarcinoma, and the highest frequency of mutated *P53* has been observed in Asian adenocarcinoma patients [26]. Moreover, differential methylation patterns between histologic subtypes were identified; hypermethylation in *PAK6* and *NOGOR* is strongly correlated with adenocarcinoma [27]. A high-throughput genotyping platform showed that squamous cell carcinoma and adenocarcinoma have distinct molecular profiles; *KRAS* mutations were identified only in adenocarcinoma and *EGFR* mutation was detected only in squamous cell carcinoma [28].

A decrease in the 5-year RSR for localized squamous cell carcinoma may be explained by the increased rates of carcinoma in situ. Increasing incidences of squamous cell carcinoma in situ, coupled with a decreasing trend in invasive squamous cell carcinoma occurrences, were documented in Korea between 1993 and 2009 [13]. This attests to the success of cervical screening and treatment of pre-invasive squamous cell carcinoma in the country. Earlier detection and treatment of carcinoma in situ may result in a decrease of microinvasive cervical cancers that progress to a localized stage, which would lead to very favorable outcomes.

This study has limitations. First, central pathology reviews were not performed for patients registered in the KCCR. Values for histologic subtypes are missing in 8.8% of the study population, and the possibility exists that some patients were misclassified. Second, the KCCR lacks data on some important demographic variables such as FIGO staging and socioeconomic status. Lastly, the KCCR lacks data on timing and distribution of recurrences.

## Conclusions

This study demonstrated that patients with adenocarcinoma have worse survival outcomes than those with squamous cell carcinoma. Considering that current management for adenocarcinoma is virtually the same as that for squamous cell carcinoma, prospective studies are warranted to evaluate histology-specific treatment protocols.

## Supporting Information

S1 Table Treatment patterns according to time period. (DOCX)
