# Supplementary material for: Prognosis of Cervical Cancer in the Era of Concurrent Chemoradiation from National Database in Korea: A Comparison between Squamous Cell Carcinoma and Adenocarcinoma
Source: PLoS One. 2015 Dec 14;10(12):e0144887. doi: 10.1371/journal.pone.0144887 (PMC4682792; doi:10.1371/journal.pone.0144887)
Supplement: S1 Table — (DOCX) [file pone.0144887.s001.docx]

**Supporting information**

S1 Table. Treatment patterns according to time period

|  |  | 1993–1997 | | 1998–2002 | | 2003–2012 | | TOTAL | |
| --- | --- | --- | --- | --- | --- | --- | --- | --- | --- |
| Stage | Primary treatment | case | % | case | % | case | % | case | % |

| Localized |  |  |  |  |  |  |  |  |  |
| --- | --- | --- | --- | --- | --- | --- | --- | --- | --- |
|  | Surgery | 3,429 | 54.2 | 4,527 | 58.1 | 12,126 | 62.2 | 20,082 | 59.8 |
|  | Chemotherapy | 512 | 8.1 | 405 | 5.2 | 620 | 3.2 | 1,537 | 4.6 |
|  | Radiation | 191 | 3.0 | 205 | 2.6 | 473 | 2.4 | 869 | 2.6 |
|  | Chemoradiation | 46 | 0.7 | 114 | 1.5 | 1,091 | 5.6 | 1,251 | 3.7 |
|  | Surgery + adjuvant chemoradiation | 81 | 1.3 | 229 | 2.9 | 1,238 | 6.4 | 1,548 | 4.6 |
|  | Others | 2,063 | 32.6 | 2,317 | 29.7 | 3,942 | 20.2 | 8,322 | 24.8 |
|  | TOTAL | 6,322 | 100.0 | 7,797 | 100.0 | 19,490 | 100.0 | 33,609 | 100.0 |
| Regional |  |  |  |  |  |  |  |  |  |
|  | Surgery | 395 | 9.6 | 444 | 9.9 | 1,391 | 15.2 | 2,230 | 12.6 |
|  | Chemotherapy | 1,094 | 26.7 | 868 | 19.3 | 588 | 6.4 | 2,550 | 14.4 |
|  | Radiation | 787 | 19.2 | 780 | 17.3 | 692 | 7.6 | 2,259 | 12.7 |
|  | Chemoradiation | 279 | 6.8 | 801 | 17.8 | 2,595 | 28.4 | 3,675 | 20.7 |
|  | Surgery + adjuvant chemoradiation | 73 | 1.8 | 255 | 5.7 | 1,810 | 19.8 | 2,138 | 12.0 |
|  | Others | 1,477 | 36.0 | 1,352 | 30.0 | 2,076 | 22.7 | 4,905 | 27.6 |
|  | TOTAL | 4,105 | 100.0 | 4,500 | 100.0 | 9,152 | 100.0 | 17,757 | 100.0 |
| Distant |  |  |  |  |  |  |  |  |  |
|  | Surgery | 23 | 8.6 | 24 | 5.8 | 125 | 5.9 | 172 | 6.2 |
|  | Chemotherapy | 51 | 19.0 | 59 | 14.1 | 263 | 12.5 | 373 | 13.4 |
|  | Radiation | 69 | 25.7 | 93 | 22.3 | 199 | 9.5 | 361 | 13.0 |
|  | Chemoradiation | 16 | 6.0 | 67 | 16.1 | 618 | 29.4 | 701 | 25.2 |
|  | Surgery + adjuvant chemoradiation | 5 | 1.9 | 15 | 3.6 | 297 | 14.1 | 317 | 11.4 |
|  | Others | 104 | 38.8 | 159 | 38.1 | 599 | 28.5 | 862 | 30.9 |
|  | TOTAL | 268 | 100.0 | 417 | 100.0 | 2,101 | 100.0 | 2,786 | 100.0 |
| Unspecified |  |  |  |  |  |  |  |  |  |
|  | Surgery | 4,185 | 38.1 | 3,605 | 39.9 | 2,738 | 41.6 | 10,528 | 39.6 |
|  | Chemotherapy | 1,387 | 12.6 | 778 | 8.6 | 308 | 4.7 | 2,473 | 9.3 |
|  | Radiation | 1,537 | 14.0 | 897 | 9.9 | 472 | 7.2 | 2,906 | 10.9 |
|  | Chemoradiation | 438 | 4.0 | 508 | 5.6 | 643 | 9.8 | 1,589 | 6.0 |
|  | Surgery + adjuvant chemoradiation | 150 | 1.4 | 249 | 2.8 | 245 | 3.7 | 644 | 2.4 |
|  | Others | 3,296 | 30.0 | 3,008 | 33.3 | 2,170 | 33.0 | 8,474 | 31.8 |
|  | TOTAL | 10,993 | 100.0 | 9,045 | 100.0 | 6,576 | 100.0 | 26,614 | 100.0 |
